# Sequence variation and regulatory variation in acetylcholinesterase genes contribute to insecticide resistance in different populations of *Leptinotarsa decemlineata*

**DOI:** 10.1002/ece3.8269

**Published:** 2021-11-01

**Authors:** Aigi Margus, Saija Piiroinen, Philipp Lehmann, Alessandro Grapputo, Leona Gilbert, Yolanda H. Chen, Leena Lindström

**Affiliations:** ^1^ Department of Biological and Environmental Science University of Jyväskylä Jyväskylä Finland; ^2^ Department of Zoology Stockholm University Stockholm Sweden; ^3^ Zoological Institute and Museum Greifswald University Greifswald Germany; ^4^ Department of Biology University of Padua Padua Italy; ^5^ Te?ted Oy Jyväskylä Finland; ^6^ Department of Plant and Soil Science University of Vermont Burlington Vermont USA

**Keywords:** carbamate, gene expression, insecticide resistance, organophosphate, target site mutation

## Abstract

Although insect herbivores are known to evolve resistance to insecticides through multiple genetic mechanisms, resistance in individual species has been assumed to follow the same mechanism. While both mutations in the target site insensitivity and increased amplification are known to contribute to insecticide resistance, little is known about the degree to which geographic populations of the same species differ at the target site in a response to insecticides. We tested structural (e.g., mutation profiles) and regulatory (e.g., the gene expression of *Ldace1* and *Ldace2*, AChE activity) differences between two populations (Vermont, USA and Belchow, Poland) of the Colorado potato beetle, *Leptinotarsa decemlineata* in their resistance to two commonly used groups of insecticides, organophosphates, and carbamates. We established that Vermont beetles were more resistant to azinphos‐methyl and carbaryl insecticides than Belchow beetles, despite a similar frequency of resistance‐associated alleles (i.e., S291G) in the *Ldace2* gene. However, the Vermont population had two additional amino acid replacements (G192S and F402Y) in the *Ldace1* gene, which were absent in the Belchow population. Moreover, the Vermont population showed higher expression of *Ldace1* and was less sensitive to AChE inhibition by azinphos‐methyl oxon than the Belchow population. Therefore, the two populations have evolved different genetic mechanisms to adapt to organophosphate and carbamate insecticides.

## Graphical abstract

We tested structural and regulatory differences between two populations of the Colorado potato beetle in their resistance to two commonly used groups of insecticides. We show that despite the similar frequency of resistance‐associated mutations in the target site gene, the more resistant populations had additional mutations in another target site gene, higher expression of the target site genes and had less sensitive to enzyme to insecticide inhibition. Therefore, the two populations have evolved different genetic mechanisms to adapt to organophosphate and carbamate insecticides.

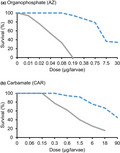

## 1 INTRODUCTION

Resistance to insecticides is a serious global problem that can negatively affect human health, food production, and agriculture (Gould et al., 2018). Since 1914, up to 597 arthropod species have become resistant to more than 300 insecticides (Roush & Tabashnik, 2012; Sparks & Nauen, 2015). The evolution of insecticide resistance is a process of genetically based decrease in population susceptibility to insecticides (IRAC, 2020). Although insecticides can have a specific selective pressure, where selection acts on a specific target, there is still considerable variation in the magnitude of insecticide resistance among the populations of the same insect species (Dively et al., 2020; Ryan et al., 2019; Wu et al., 1999). These differences have often been linked to the genetic differences at the target site (Ilias et al., 2014; Weill et al., 2003). Despite increasing knowledge of the biochemical basis of insecticide resistance, relatively little is known about the geographic difference in insecticide target site gene sequences or their regulation (Hawkins et al., 2019; Ryan et al., 2019).

Pest species can become resistant to insecticides via various structural and regulatory mechanisms (Feyereisen et al., 2015). Structural mechanisms are better characterized and involve target site mutations in an enzyme that make the insecticide ineffective. These can be produced with genetic differences such as nonsynonymous nucleotide variation at target site genes (Li & Han, 2004; Malekmohammadi & Galehdari, 2016; Weill et al., 2004; Zhu et al., 1996). Regulatory mechanisms have been less studied and can be related, for example, to biochemical processes such as the overexpression of the target site or enhanced metabolism or excretion of the insecticide (Barres et al., 2016; Ffrench‐Constant, 2013). In general, we know less about the variation among populations at both the nucleotide and regulatory levels of the target site genes. By understanding population‐level variation also at the regulatory level, we may be able to explain better why the same insecticide might cause different outcomes among populations. This is because our predictions based on gene sequence variation alone might bias our estimates of the overall pesticide resistance (see discussion in Hawkins et al., 2019).

Organophosphates (OPs) and carbamates are both widely known as acetylcholinesterase (AChE; EC 3.1.1.7) inhibitors, which have been heavily used as insecticides since the 1970s. The AChE enzyme functions at the synapses of cholinergic neurons in the central and peripheral nervous system (Fukuto, 1990; Taylor, 2011; Taylor et al., 2009). The enzyme terminates neurotransmission at cholinergic synapses in the synaptic cleft by hydrolyzing the neurotransmitter acetylcholine (Taylor et al., 2009). OP and carbamate insecticides inhibit AChE, thus interfering with neurotransmission, leading to paralysis and death (Colovic et al., 2013). In many invertebrates, there are two distinct acetylcholinesterases (AChE1 and AChE2), which are encoded by two *ace* genes (i.e., *ace1* and *ace2*; Kim & Lee, 2013). The two AChEs are probably homologous, derived from an ancient duplication event that occurred long before the differentiation of insects (Weill et al., 2002). In 67 insect species out of 100, AChE1 accounts for most of the AChE activity and thus is considered as the main catalytic enzyme (Kim & Lee, 2013) responsible for neuronal functions (Weill et al., 2002). However, the role of AChE2 differs between species. It can act as the main catalytic activity, be equally active to AChE1, or show little catalytic activity (Kim & Lee, 2013). For example, in *Bombyx mori*, *Bm*‐*ace2* is more highly expressed and thus likely the main catalytic enzyme, while *Bm*‐*ace1* may contribute to metamorphosis (Chen et al., 2009). However, more functional studies are needed to demonstrate the exact functions of these two genes (Jiang et al., 2018).

The Colorado potato beetle (CPB), *Leptinotarsa decemlineata* Say (Coleoptera: Chrysomelidae), is a model organism for studying the evolution of insecticide resistance (Schoville et al., 2018). CPB has played an important role in the modern pesticide industry. Since it was first targeted by insecticides in 1864, it has been heavily managed with insecticides (Alyokhin et al., 2008; Gauthier et al., 1981). CPB has currently developed resistance to more than 50 different active ingredients used in insecticides, including OPs (16 active ingredients) and carbamates (five active ingredients; Alyokhin et al., 2008; Brevik et al., 2018; Mota‐Sanchez & Wise, 2020). CPB populations in North America and Europe have developed resistance to both classes of insecticides (Mota‐Sanchez & Wise, 2020). Until recently, only one *ace* gene, *Ldace2*, orthologous to the *Drosophila melanogaster ace2* gene (Zhu & Clark, 1995), has been associated with the resistance to OP and carbamate insecticides in CPB (Zhu et al., 1996). Three mutations (S291G, R30K, and Y45H) in *Ldace2* gene have been described and linked to OP and/or carbamate resistance (Kim et al., 2007; Zhu & Clark, 1997; Zhu et al., 1996), with the S291G mutation being the main target site conferring resistance (Zhu et al., 1996). Revuelta et al. (2011) described *Ldace1* for the CPB which is an orthologous to the *Anopheles gambiae ace1* gene. The authors suggested that the 2‐ to 11‐fold higher expression of *Ldace1* compared with *Ldace2* indicated that *Ldace1* rather than *Ldace2* was the main contributor to the AChE activity and may have been the primary target of the OP insecticides (Revuelta et al., 2011).

We set to study whether the differences among populations in mortality are linked to sequence variation and/or regulatory resistance mechanisms. We hypothesized that sequence and regulatory variation in *Ldace1* and *Ldace2* genes (but see Piiroinen et al., 2013) might contribute to differences in OP and carbamate resistance development in different geographic populations. We tested how the *Ldace1* and *Ldace2* genes contribute to resistance to OP and carbamate insecticides by comparing CPB populations that differ in their resistance to these insecticides. Using bioassays, we started by estimating the resistance level of six different populations to the organophosphate azinphos‐methyl (AZ) and the carbamate carbaryl (CAR) insecticides. Based on the resistance status, we selected two populations, Vermont (from the USA) as the most resistant, and Belchow (from Poland) as the less resistant population, for further investigation of *Ldace1* and *Ldace2* genes. To test for population‐level differences, we first measured the efficacy of AChE inhibition by azinphos‐methyl oxon (AZoxon) and CAR insecticides. Then, we measured *Ldace1* and *Ldace2* gene expression, both before exposure and after exposure to both insecticides. Finally, we sequenced the *Ldace1* and *Ldace2* genes and investigated the frequency of amino acid replacements (mutations) in the two populations. We predicted that the more resistant population (i) would be less sensitive to AChE insecticide inhibition, (ii) demonstrate higher target site expression levels, and (iii) show higher frequency of mutations at target sites.

## 2 MATERIALS AND METHODS

### 2.1 Study species and rearing conditions

Colorado potato beetles used in this study were descendants of beetles collected from potato fields from Vermont, USA (44°43′N, 73°20′W), Belchow, Poland (52°01′N, 20°34′E), Padua, Italy (45°48′N, 12°07′E), Emmen, the Netherlands (52°54′N, 6°51′E) in 2010, near Ufa, Russia (54°47′N, 55°57′E) in 2009, and Petroskoi, Russia (61°49′N, 34°10′E) in 2006. The experiments were performed in 2012, and thus, the populations had been reared without exposure to insecticide for 2–6 generations (one generation/year). In each generation, unrelated parental beetles (i.e., overwintered generation) were mated within the population and each pair (i.e., family) was reared in a petri dish lined with a moisturized filter paper and fed daily with fresh potato leaves (*Solanum tuberosum* variety Van Gogh). To maintain genetic diversity in the laboratory stock, at least 50 families were reared in each generation. Eggs were collected daily, and larvae were reared in family groups until adulthood. Beetles were maintained at a constant temperature of 23°C under a fluctuating light regime of 18‐h light (16‐h light with 1‐h dim light imitating sunset and sunrise) and 6‐h dark in controlled environmental chambers (Type B1300, Weiss Technic). After rearing the beetles for one generation with an 18:6 L:D photoperiod, we simulated fall conditions to ensure that all populations induced diapause, and we reared newly emerged adult beetles under a 12:12 L:D photoperiod (Lehmann et al., 2014a). Adult beetles of the summer generation overwintered individually at 5°C in environmental chambers.

### 2.2 Insecticide bioassays

We used bioassays to determine whether the different geographic populations differ in the degree of insecticide resistance to azinphos‐methyl (AZ; organophosphate) and the carbaryl (CAR; carbamate) insecticides (Ovčarenko et al., 2014). For the bioassays, we obtained both AZ and CAR as Pestanal analytical standards from Sigma‐Aldrich, which were dissolved in acetone. Bioassays were performed by applying insecticide treatment to early third instar (5–7 days old, Bointeau & Le Blanc, 1992) larvae using a pipette. Larvae were randomly divided into petri dishes (five individuals/family/petri dish/) and were randomly assigned to an insecticide treatment. Then, we applied a 3 µl drop of AZ, CAR, or acetone (as control) topically to the fifth and sixth dorsal abdominal segments. In order to calculate the median lethal dose (LD_50_), we treated each of the six populations (*N* = 160–414 larvae) with 4–8 different insecticide doses ranging from 0.0075 to 30 µg/larvae for AZ and 0.06 to 90 µg/larvae for CAR (3–6 larvae per family, 20–74 larvae per insecticide dose, see Figure S1). After the insecticide application, we provided larvae a standardized potato leaf after 2 h and assessed survival after 22 h (24 h of survival from the application). A larva was considered dead if it was not able to move after being placed on its back.

We analyzed larval survival separately for both insecticides using generalized linear models (GLM; Binary logistic, logit link function) in SPSS (IBM SPSS Statistics 24.0.0). Population and insecticide dose were set as explanatory variables and survival as the dependent variable. We determined the resistance levels (LD_50_, i.e., lethal dose) and generated the 95% confidence limits for each population and insecticide using Probit analysis. We considered differences in LD_50_ values between populations and insecticides significant if their 95% confidence limits did not overlap (Ovčarenko et al., 2014). No mortality was observed in any of the populations within the acetone control.

### 2.3 Sampling AChE enzyme activity and gene expression studies

**FIGURE 1:**
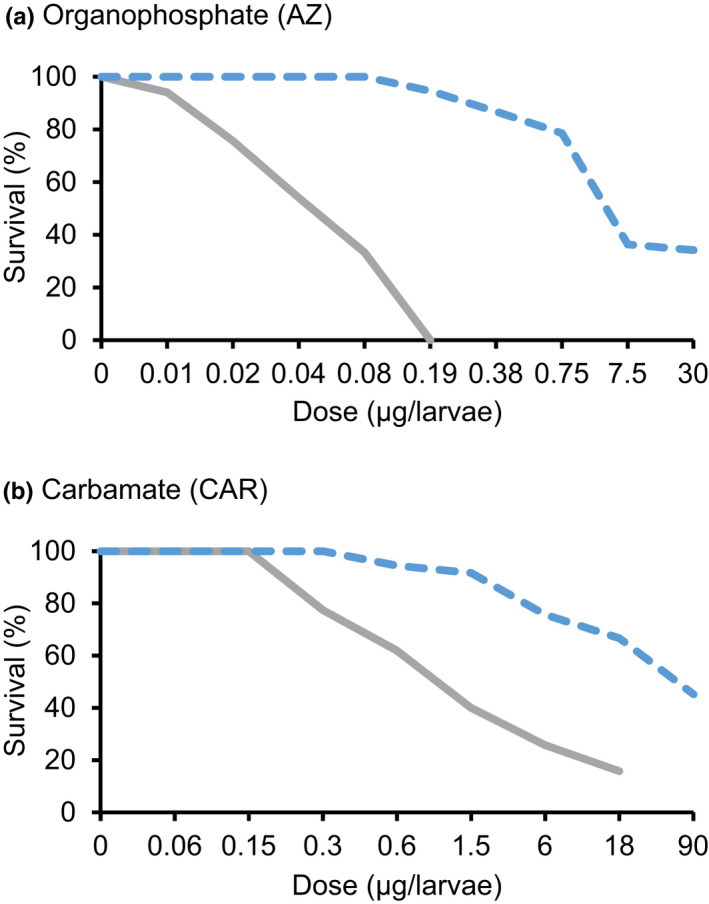
Survival (%) of Colorado potato beetles from Belchow (gray solid line) and Vermont populations (blue dashed line) after exposure to different doses (µg/larvae) of (a) azinphos‐methyl (AZ) and (b) carbaryl (CAR) insecticides

Based on bioassay results, the Vermont and Belchow populations were selected for further studies to investigate the AChE activity, gene expression, and nonsynonymous point mutations in the two genes (Tables 1, 2; Figure 1). We selected the Vermont population because it showed the highest tolerance to both insecticides, while the Belchow population was less resistant. Since the LD_10_ dose of Vermont population would have killed all the beetles from Belchow population, we applied population‐specific LD_10_ doses to cause 10% mortality (LD_10_) and induce a similar level of insecticidal stress. We used the same protocol described above for applying insecticide doses of AZ (Vermont 0.375 µg/larvae and Belchow 0.0075 µg/larvae), CAR (Vermont 1.5 µg/larvae and Belchow 0.15 µg/larvae), and acetone as control. After the insecticide application, we collected the surviving larvae 2, 4, and 24 h from the insecticide application, froze them in liquid nitrogen, and stored at −80°C until further analysis. In total, we collected 713 larvae from 15 Vermont families and 417 larvae from 15 Belchow families.

**TABLE 1:** AZ insecticide toxicity to the Colorado potato beetle larvae

Population	*N*	Slope (±SE)	*Z*	*p*	LD_50_ (µg/larvae) (95% confidence limits)	Resistance ratio (RR)^a^
Ufa (Russia)	160	60.06 (±10.11)	5.94	<.001	0.02 (0.006–0.045)	1
Belchow (Poland)	199	26.94 (±4.26)	6.32	<.001	0.04 (0.017–0.086)	2.3
Petroskoi (Russia)	232	21.53 (±2.93)	7.35	<.001	0.05 (0.024–0.113)	3.0
Emmen (Netherlands)	205	6.20 (±0.79)	7.81	<.001	0.05 (0.021–0.107)	2.9
Padua (Italy)	223	0.22 (±0.04)	5.16	<.001	0.22 (0.099–0.476)	13.1
Vermont (USA)	322	0.06 (±0.01)	7.48	<.001	4.14 (1.998–9.416)	246.1

Note

LD_50_ (i.e., lethal dose that kills 50% of exposed individuals within 24 h since exposure) values for the six Colorado potato beetle populations exposed to the AZ insecticide.

^a^ Resistance ratio showing the increase in resistance compared to the least (Ufa) resistant population.

**TABLE 2:** CAR insecticide toxicity to the Colorado potato beetle larvae

Population	*N*	Slope (±SE)	*Z*	*p*	LD_50_ (µg/larvae) (95% confidence limits)	Resistance ratio (RR)^a^
Ufa (Russia)	231	0.488 (±0.084)	5.83	<.001	0.33 (0.155–0.705)	1
Belchow (Poland)	267	0.438 (±0.056)	7.83	<.001	0.41 (0.199–0.845)	1.2
Petroskoi (Russia)	256	0.109 (±0.014)	7.99	<.001	2.15 (1.065–4.395)	6.5
Emmen (Netherlands)	414	0.017 (±0.003)	6.51	<.001	3.70 (2.064–6.974)	11.2
Padua (Italy)	211	0.012 (±0.003)	4.29	<.001	71.39 (29.831–187.697)	215.1
Vermont (USA)	286	0.017 (±0.003)	6.55	<.001	43.23 (19.496–104.350)	130.3

Note

LD_50_ values for the six Colorado potato beetle populations exposed to the CAR insecticide.

^a^ Resistance ratio showing the increase in resistance compared with the least (Ufa) resistant population.

### 2.4 Measurement of AChE inhibition efficiency

We tested *in vitro* whether populations that can tolerate higher insecticide doses (see Tables 1, 2) possess an AChE variant that is less sensitive to insecticide inhibition by using a modified Ellman et al. (1961) protocol (see also Anderson & Coats, 2012; Pang et al., 2012; Singh et al., 2017). We assessed AChE enzyme activity inhibition from the control treatment larvae; that is, treated with only acetone, the 2‐ and 4‐h groups (*n* = 25 and *n* = 28 for Vermont and Belchow, respectively). Individual larvae were put into 200 µl of ice‐cold 1xPBS buffer (pH 7.5) containing 0.5% (v/v) Triton X‐100, homogenized by crushing for 2–3 min, and sonicated for 40 s before centrifuging at 15,871 *g* for 20 min at 4 °C. The total protein concentration was measured from the supernatant with a NanoDrop ND‐1000 spectrophotometer (NanoDrop Technologies, Wilmington, USA). Thereafter, samples were normalized to a concentration of 15 mg/ml. The supernatants were subsequently used for the enzyme activity inhibition analysis. Enzyme activity was determined by an Ellman et al. (1961) method with the QuantiChrom™ Acetylcholinesterase Assay Kit (DACE‐100; BioAssay Systems) following the manufacturer’s instructions. The reaction mixture for inhibition consisted of 15 µl of supernatant and 2 µl of the AChE enzyme inhibitor (12.9 and 250 µM for azinphos‐methyl oxon (AZoxon, Sigma‐Aldrich) and CAR, respectively). We used AZoxon in this assay because AZ is metabolized to AZoxon when an insect ingests it. Insecticide stock solutions (AZoxon and CAR in 100% EtOH) were first diluted with water so that the final concentration of EtOH in the inhibition reaction was less than 1%. After the inhibition reaction was incubated for 2 min at RT, the supernatant and inhibitor solution were added to 190 µl of the working reagent provided by the manufacturer, after which the absorbance was measured 2 and 10 min later at 405 nm with a Victor X4 2030 multilabel plate reader (PerkinElmer). Each sample was measured with three technical replicates. AChE enzyme activities were determined following the manufacturer’s instructions. AChE activity after inhibition was calculated as the percentage of enzyme activity after insecticide inhibition divided by enzyme activity without inhibition. We tested whether the populations differed in AChE activity after insecticide inhibition (%) was analyzed using separate one‐way ANOVA tests in SPSS version 26.0 (IBM SPSS Statistics) for each insecticide. We used Levene’s test to test for homogeneity of variance and Shapiro–Wilk test to test for normality.

### 2.5 Expression of AChE genes

We tested for differences in the gene expression level of *Ldace1* and *Ldace2* between Vermont and Belchow populations. We sampled larvae 24 h after the treatment applications. Total RNA was extracted from individual larvae with the TriReagent (Sigma‐Aldrich) and the RNeasy Mini RNA Extraction Kit (Qiagen). Extraction was followed by a clean‐up step using RNeasy columns, including DNase I treatment (RNase‐Free DNase Set, Qiagen). The concentration and purity of RNA was measured with a NanoDrop ND‐1000 spectrophotometer. RNA integrity and quality were checked with an Agilent 2100 Bioanalyzer (Agilent). The RNA concentration was normalized to 100 ng/µl before generating complementary DNA (cDNA) using the iScript™ cDNA Synthesis Kit with oligo (dT) and random hexamer primers (Bio‐Rad Laboratories Inc.). The qPCR reaction mix contained 10 µl of 2× SYBR Green Supermix (Bio‐Rad Laboratories Inc.), 0.5 µM of each gene‐specific primer, and 5 µl of cDNA (diluted 1:4) for a total volume of 20 µl. We designed primers used for qPCR (Table S1) amplification of *Ldace1* (JF343436.1; (Revuelta et al., 2011)) and *Ldace2* (L41180.1; (Zhu & Clark, 1995)) using Primer 3 (http://frodo.wi.mit.edu/primer3/, v. 0.4.0) according to Lehmann, Piiroinen, et al. (2014). The primers were designed based on sequences from the annotated transcriptome of CPB (Kumar et al., 2014) and sequences available in GenBank. The qPCRs were run on a Bio‐Rad CFX96™ instrument with an initial denaturation step of 95°C for 3 min, followed by 39 cycles of 10 s at 95°C, 10 s at 56°C, and 30 s at 72°C. The qPCR was followed by melting curve analysis (65–95°C) to check the purity of qPCR. We analyzed ten biological replicates (individual beetles) with three technical replicates for each treatment group (control, AZ, and CAR) for each population. In order to calibrate the expression across samples, we used two positive control samples with two replicates that were added to each plate. The efficiency of qPCR amplification was calculated for each gene using twofold serial dilutions of pooled cDNA. The amplification efficiencies were between 96% and 113%.

We calculated expression values (mean Cq) for all the samples using the normalized expression (ΔΔCq) method with default threshold values by using the CFX Manager 3.0 software (Bio‐Rad Laboratories Inc.). We used forkhead transcription factor (*FOXO*) and ribosomal protein L13e (*L13e*) as reference genes (Table S1; Kumar et al., 2014; Lehmann, Piiroinen, et al., 2014; Yocum et al., 2009). We analyzed the relative expression data with the REST program (http://rest.gene‐quantification.info/; Pfaffl et al., 2002), according to Lehmann, Piiroinen, et al. (2014). The REST program (with 10,000 iterations) was used for pairwise comparisons within and between population and treatment groups.

### 2.6 Identification of sequence variation in the Ldace1 and Ldace2 genes

To identify and compare mutations in *Ldace1* and *Ldace2* between populations, we sequenced the genes from the experimental larvae. We extracted total RNA from 38 (Belchow *n* = 19, Vermont *n* = 19) whole larvae and synthesized cDNA using the same procedures described earlier. Primers for *Ldace1* and *Ldace2* (Table S2) were designed using the CPB sequences available in GenBank [*Ldace1*: JF343436.1, (Revuelta et al., 2011); *Ldace2*: L41180.1, (Zhu & Clark, 1995)] similarly as described before. PCRs were performed in a 25 µl reaction containing 4 µl of cDNA, 1× of Dream buffer (Thermo Fisher Scientific), 1 µM of forward and reverse primer, 0.2 mM of dNTPs, and 0.2 U of Dream Taq DNA polymerase (Thermo Fisher Scientific). The cycling conditions for the PCR were 94°C for 3 min, then 35 cycles at 94°C for 45 s, 56°C for 1 min 30 s, and 72°C for 1 min 30 s. PCR products were purified using Exonuclease (Exonuclease I, Thermo Fisher Scientific) and Shrimp Alkaline Phosphatase (SAP, Thermo Fisher Scientific). Sequencing reactions were performed using BigDye^®^ Terminator v3.1 Cycle Sequencing Kit (Thermo Fisher Scientific). Sequencing reactions were performed in a 20 µl reaction containing 3.75 µl of 5× BigDye Sequencing buffer, 0.5 µl of 2.5× Ready Reaction Premix, 1 µl of 3.2 µM of the primer, and 3–10 µl of the purified PCR product. Thereafter, samples were run on an ABI Prism 3130xl genetic analyzer (Thermo Fisher Scientific). Chromatograms were analyzed using Geneious version 8.1.9 (Biomatters, Ltd, New Zealand, http://geneious.com (Kearse et al., 2012)). The 1036 bp of *Ldace1* and 1890 bp of *Ldace2* were aligned by codons with the reference sequence from GenBank, *Ldace1* (JF343436.1; Revuelta et al., 2011) and *Ldace2* (L41180.1; Zhu & Clark, 1995) using Muscle in Geneious with default parameters. In total, we analyzed the sequences of 36 (Belchow *n* = 17, Vermont *n* = 19) and 37 (Belchow *n* = 19, Vermont *n* = 18) individuals for *Ldace1* and *Ldace2*, respectively. Frequency of amino acid replacements was compared between populations by a chi‐square test in SPSS.

## 3 RESULTS

### 3.1 Insecticide bioassays

Beetle survival (i.e., insecticide resistance) varied among all six populations for both insecticides, AZ (Wald χ^2^ = 742.1, df = 5, *p* < .001; Table 1) and CAR (Wald χ^2^ = 965.3, df = 5, *p* < .001; Table 2, Figures 1 and S1). Survival decreased with increasing dose of AZ (Wald χ^2^ = 930.1, df = 8, *p* < .001) and CAR insecticides (Wald χ^2^ = 1650.0, df = 7, *p* < .001). There was a significant interaction between the insecticide dose and population: AZ (Wald χ^2^ = 8408.0, df = 15, *p* < .001) and CAR (Wald χ^2^ = 5253.8, df = 20, *p* < .001). These significant interactions indicated that different populations tolerated different doses of insecticides, which can be seen as differences in the LD_50_ values among populations (Tables 1, 2). The lowest LD_50_ values to AZ insecticide were recorded for Ufa, Belchow, Petroskoi, and Emmen, whereas the highest values were recorded for Vermont (Table 1). The lowest LD_50_ values for the CAR insecticide were recorded in Ufa and Petroskoi, whereas the highest values were in Vermont and Padua (Table 2). The Vermont population was 107—times more resistant to AZ and 20—times more resistant to CAR insecticide than the Belchow population when comparing the LD_50_ levels.

### 3.2 Insecticide inhibition efficiency

**FIGURE 2:**
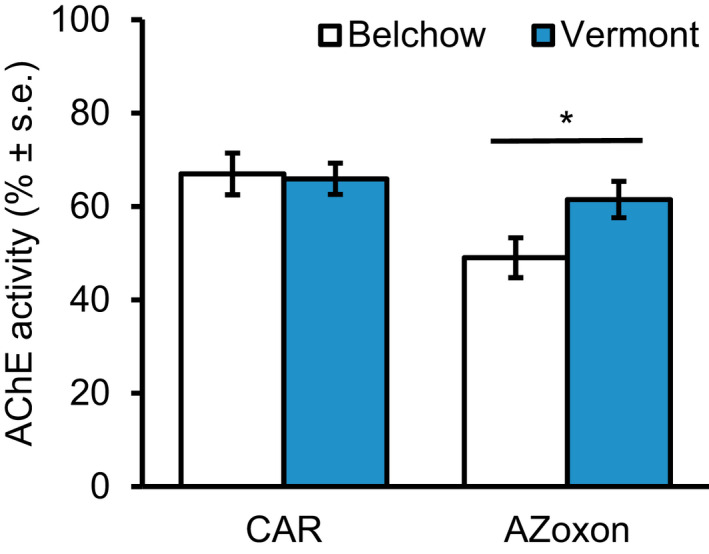
AChE activity (% ± s.e.) after inhibition with either a) 250 µM of carbaryl (CAR) or (b) 12.9 µM of azinphos‐methyl oxon (AZoxon) of Colorado potato beetles from Belchow (white) and Vermont (blue) populations. * indicates significant difference (*p* < .05) between the populations

Both insecticides inhibited AChE activity by more than 30% (CAR: *t* test =8.6, df = 24, *p* < .001, AZoxon: *t* test =10.8, df = 36, *p* < .001; Figure 2). However, the Belchow population was more sensitive to AZoxon inhibition than the Vermont population (*F*_1,35_ = 11.6, *p* = .002). The AChE activity in Belchow beetles was 12% more inhibited than those in Vermont beetles (*F*_1,35_ = 4.6, *p* = .039; Figure 2). In contrast, there were no differences between the two populations in AChE activity after inhibition by CAR (*F*_1,23_ = 0.034, *p* = .855; Figure 2).

### 3.3 Expression of AChE genes

**FIGURE 3:**
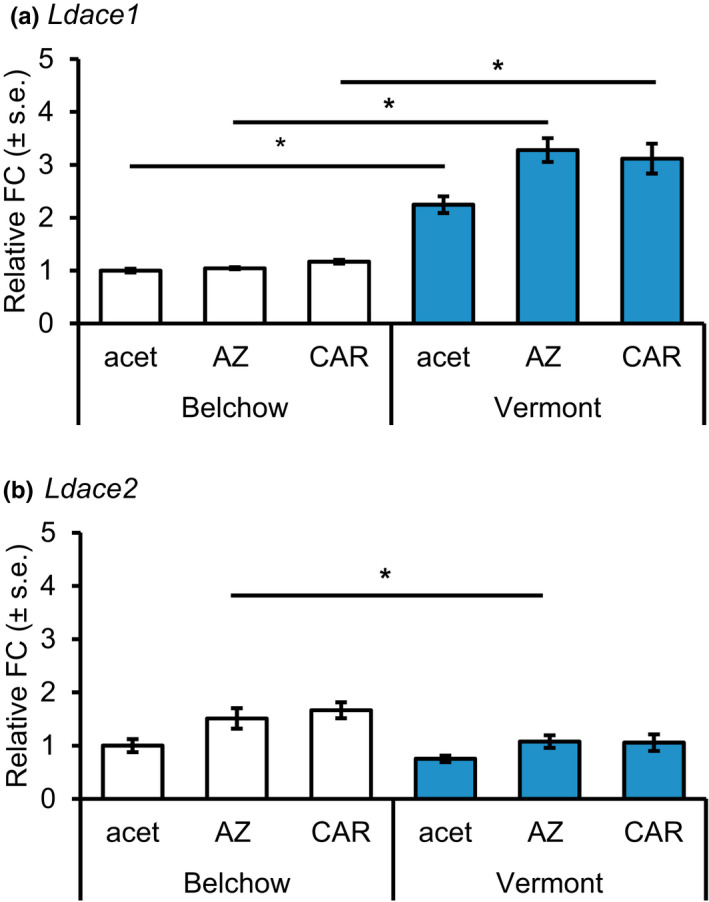
Relative fold change (FC ± s.e.) to Belchow control (acet) group of (a) *Ldace1* and (b) *Ldace2* expression levels between different treatments azinphos‐methyl (AZ), carbaryl (CAR), and control (acet) for Colorado potato beetles from Belchow (white) and Vermont (blue) populations. The fold change difference between *Ldace1* and *Ldace2* transcripts was 49 in the Belchow population and 145 in the Vermont population. * indicates a significant difference (*p* < .05) between populations

The *Ldace1* gene was expressed at a higher level in the beetles than the *Ldace2* gene in both Belchow (Fold change (FC = 49, *p* < .001) and Vermont (FC = 145, *p* < .001) populations (Figure 3). The expression of *Ldace1* significantly differed between populations (Figure 3a). *Ldace1* was significantly upregulated in all treatment groups in the Vermont population compared to the Belchow population (Figure 3a). Averaged across all treatment groups, *Ldace1* expression was 2.4‐fold higher in the Vermont population than in the Belchow population. The differences in expression varied significantly between populations for each treatment group: control group (FC = 2.3, *p* < .001), AZ group (FC = 2.5, *p* < .001), and CAR group (FC = 2.4, *p* = .01; Figure 3a). Within‐population comparisons revealed that exposure to insecticide (both to AZ and CAR) did not induce changes in *Ldace1* expression levels when compared to the control group (Figure 3a).

*Ldace2* was not statistically differently expressed in the Vermont and Belchow populations in both the control and CAR insecticide groups (Figure 3b.). However, exposure to the AZ insecticide resulted in downregulation of *Ldace2* gene in Vermont compared with the Belchow population (FC = 0.85, *p* = .04). Within the Belchow population, *Ldace2* expression was marginally non‐significantly (FC = 1.7, *p* = .059) upregulated in the AZ exposed group when compared to the control group. This suggested that individuals in the Belchow population increased *Ldace2* expression in response to AZ insecticide exposure. We did not identify any between‐ or within‐population effects on *Ldace2* expression when comparing the CAR insecticide treatment group to the control group.

### 3.4 Identification of sequence variation in the Ldace1 and Ldace2 genes

**FIGURE 4:**
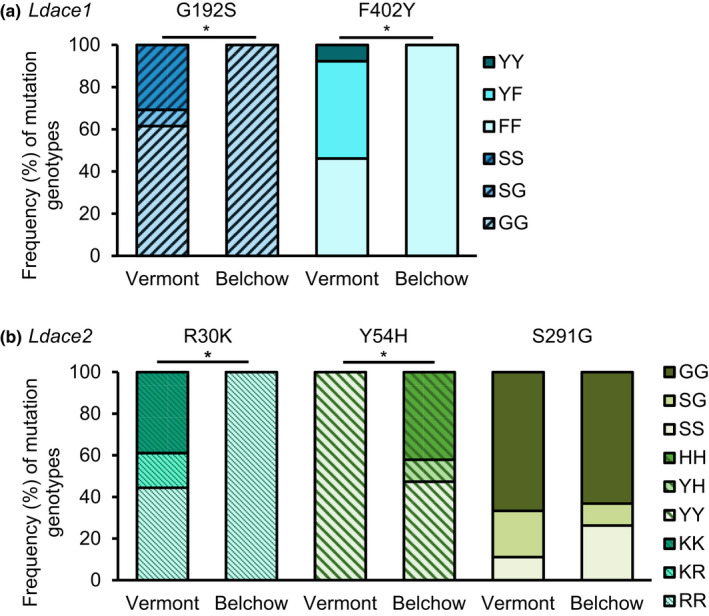
Frequency (%) of different genotypes for alleles in the (a) *Ldace1* and (b) *Ldace2* gene in the Colorado potato beetle. Light shades show the frequency of homozygous non‐mutated sites, intermediate shades show the frequency of heterozygous sites, and dark shades show homozygous mutated sites. * indicates significant difference (*p* < .05) between populations. SS‐homozygous for G192S allele, SG‐heterozygous, GG‐homozygous, lacking G192S allele; YY‐homozygous for the F402 allele, YF‐heterozygous, FF‐homozygous, lacking the F402 allele in the *Ldace1* gene. KK‐homozygous for R30K allele, RK‐heterozygous, RR‐homozygous lacking R30K allele; HH‐homozygous for Y54H allele, YH‐heterozygous, YY‐lacking the Y54H allele; GG‐homozygous for the OP resistance‐associated allele S291G, SG‐heterozygous, SS‐homozygous, lacking the S291G allele in the *Ldace2* gene

Sequencing analyses revealed five nonsynonymous mutations in the AChE genes: G192S and Y402F in the *Ldace1* gene and R30K, Y54H, and S291G in the *Ldace2* gene. In the *Ldace1* gene, the G192S and Y402F mutations were only present in the Vermont population (Figure 4a). Allele frequencies differed significantly (G192S: χ^2^ = 7.8, df = 2, *p* = .020; Y402F: χ^2^ = 10.7, df = 2, *p* = .005; Figure 4b) between populations. Homozygotes for the G192S and Y402F mutations represented 31% and 8% of the individuals from the Vermont population, respectively. The two alleles never co‐occurred in the same individual.

In the *Ldace2* gene, the R30K mutation was only present in the individuals from the Vermont population, whereas the Y54H mutation was only present in the Belchow population. The S291G mutation was identified in both populations (Table 2; Figure 4b). Allele frequencies of the R30K and Y54H mutations differed significantly between the populations (R30K: χ^2^ = 14.5, df = 2, *p* = .001; Y54H: χ^2^ = 13.0, df = 2, *p* = .002; Figure 4b). In the Vermont population, the R30K and S291G mutations were present in 39% and 67% of the individuals, respectively. In the Belchow population, the frequency of the Y54H mutation was 42%, whereas that of the S291G mutation was 63% (Figure 4b). The frequency of the S291G mutation was similar in both populations (S291G: χ^2^ = 1.9, df = 2, *p* = .382). Both the R30K (in Vermont) and the Y54H (in Belchow) alleles occurred together with the S291G in 39% and 42% of individuals, respectively.

## 4 DISCUSSION

We investigated the population‐level sequence variation of resistance‐associated *Ldace1* and *Ldace2* genes together with their responses to two commonly used pesticides against the Colorado potato beetle to understand the role of these genes on insecticide resistance. We found that geographic populations differ in their resistance to two commonly used insecticides due to multiple differences in the insecticide target site. We demonstrated that the North American population (Vermont, USA) had higher resistance compared with a European population (Belchow, Poland), which was associated with higher occurrence of mutations (Figure 4), higher baseline expression of the *Ldace1* gene (Figure 3a), and an AChE enzyme less sensitive to AZoxon insecticide inhibition (Figure 2). In addition, the Vermont population had two mutations in the *Ldace1* gene that were absent from Belchow (Figure 4a). Therefore, it is likely that repeated application of insecticides (Dively et al., 2020) or different insecticide intensities (Crossley et al., 2018) together with invasion history (Grapputo et al., 2005) has resulted in the evolution of sequence variation and regulatory changes in the target genes that probably contributed to the overall higher resistance of the Vermont population (Figure 1).

We identified two novel mutations (G192S and F402Y) in the *Ldace1* gene in the Vermont population. The absence of these mutations in the Belchow population could be due to the loss of genetic variation when the beetle invaded Europe (Grapputo et al., 2005). Although the mutation in the *Ldace2* S291G site has been previously described as the main mutation contributing to organophosphate insecticide resistance in the CPB (Zhu & Clark, 1997; Zhu et al., 1996), the fact that it was equally common in both Vermont and Belchow populations suggests that it is unlikely to be the main factor explaining explains the 107‐time difference in survival between the populations (Figure 1). It may be possible that the ancestral state of the resistance mutation in the *Ldace2* gene has been G291S rather than S291G. This is because individuals with the resistance mutation are more susceptible to the host plant alkaloids (Wierenga & Hollingworth, 1992). This mutation might be related to the beetles’ adaptation to the lower concentrations of steroidal alkaloids in agricultural solanaceous plants rather than insecticide resistance (Piiroinen et al., 2013; Wierenga & Hollingworth, 1992; Zhu & Clark, 1995). Therefore, it is possible that the novel mutations in the *Ldace1* gene might play a more important role in the insecticide resistance than the resistance‐associated mutation S291G in the *Ldace2* gene.

The two novel mutations (see Figure 4a) described here could contribute more to the insecticide resistance in the Vermont population than the previously identified mutations in the *Ldace2* gene. Similar substitutions in the *ace1* gene (glycine to serine (G192S) and phenylalanine to tyrosine (F402Y)) have been associated with insecticide resistance (e.g., insensitivity) in other insects. Different substitutions in the *ace*‐*1* gene occur frequently across multiple species, for example, the glycine to serine substitution (i.e., G119S) in *Anopheles gambiae* (Weetman et al., 2015) and *Culex pipiens* (Weill et al., 2003, 2004), phenylalanine to tyrosine (i.e., F327Y, F331Y, and F445Y) in *Musca domestica*, *Bemisia tabaci*, and *Culex tritaeniorhnchus* (Alon et al., 2008; Nabeshima et al., 2004; Oh et al., 2006; Walsh et al., 2001). Some of the mutations in the *ace1* seemed to confer high levels of resistance in combination with other mutations in other species (Mutero et al., 1994; Vontas et al., 2002) but interestingly, in our sample, the two mutations (G192S and F402Y) never co‐occurred in *Ldace1*. We would need more functional studies to confirm the role these mutations play the insecticide resistance of the Colorado potato beetle.

In addition to substitution differences, we also identified gene expression differences between *Ldace1* and *Ldace2* in the two populations (see also Dively et al., 2020). The *Ldace1* gene was 49‐ to 145‐fold more expressed than *Ldace2* under control conditions, suggesting that *ace1* encoding ACHE1 is the major catalytic enzyme also in the CPB. These results are consistent with previous data, which indicated that in 66 insect species out of 100, AChE1 is more highly expressed than AChE2 and therefore the major catalytic enzyme of acetylcholine (Kim & Lee, 2013). Compared to a previous study (Revuelta et al., 2011), the difference between the *Ldace1* and *Ldace2* gene expression within Belchow (49‐fold difference) and Vermont (145‐fold difference) populations was higher than the reported difference between the developmental stages (i.e., from embryos to adults, 2‐ to 11‐fold difference). The increased expression in the Vermont beetles suggests that the *Ldace1* gene could also play a role in insecticide resistance. Indeed, regulatory changes in the target gene (i.e., increased expression of acetylcholinesterase gene) have been previously shown to increase OP resistance in the greenbug (*Shizaphis graminum*; Gao & Zhu, 2002). The lack of differences in the *Ldace2* expression between populations in the control groups further suggests a greater role for *Ldace1* than *Ldace2* in conferring resistance to insecticides in the CPB.

The Vermont population (see Figure 1) was less sensitive to AZoxon inhibition than the Belchow population. These differences could be explained either by regulatory resistance, that is, the lower gene expression of the Belchow populations compared with the Vermont population, or alternatively by structural resistance, that is, the mutation profile differences between the populations. Unfortunately, our sampling does not allow us to separate these two hypotheses. Previously, AChE sensitivity in the CPB has been associated with S291G and R30K mutations in the *Ldace2* gene (Kim et al., 2006), but they were not aware of the presence of the *Ldace1* gene. Therefore, it would be interesting to test the sensitivity differences related to the two mutations in the *Ldace1* (G192S and F402Y) instead. Although the CAR insecticide inhibited AChE, there were no differences between populations. This suggests that alternative resistance mechanisms are present since there was still 20‐time survival difference between the two populations. Alternatively, the differences between populations could be due to differences in OP and carbamate insecticide‐induced inhibitory actions (Colovic et al., 2013). Finally, we cannot entirely exclude an insufficient dose in the enzyme inhibition assay.

Our results demonstrate that CPB resistance to commonly used OP and carbamate insecticides is due to multiple mechanisms acting at one target site that are not mutually exclusive. Besides changes at these target sites, it is likely that other resistance mechanisms have also been under selection (see Barres et al., 2016; Mutero et al., 1994), so we cannot expect that geographically separated populations have similar mechanisms of resistance. Therefore, the same management strategy in vast areas may not be equally successful across different populations and may potentially select for further differences among populations. Differences in the resistance mechanism can also challenge the development of new application strategies if the resistance of a population differs more in structural (has higher genetic variation) or regulatory (is better able to deal with xenobiotics) mechanisms. Further research is clearly needed to uncover which mechanisms confer the resistance in different populations. Understanding the mechanisms behind the rapid evolution of insecticide resistance will help us in making better insecticide risk assessment and management strategies, in this and other pest species.

## 5 CONCLUSIONS

Our results indicate that the differences we observe in insecticide resistance among populations are a result of multiple factors acting at the same time. We demonstrated that within the target site, we need to incorporate both sequence variation and regulatory changes in AChE biochemistry and physiology to understand resistance evolution. The fact that populations differ in multiple levels even within one target site means that populations can respond to the same selection pressure in different ways. From a management perspective, it is important to understand that insecticides select not only for resistance genes but also for their function at the same time. Beetles from the Vermont population had (1) an AChE that is less sensitive to insecticide inhibition, (2) higher *Ldace1* gene expression (which suggests that AChE1 is the major catalytic enzyme in the CPB), and (3) more target site mutations (G192S and F402Y) in the *Ldace1* gene than the Belchow population. At the same time, our results underlined that studying changes at the target sites were not sufficient in explaining the observed resistance differences between populations and that there are other pathways to achieve the resistance besides changes at the target sites (see Barres et al., 2016). Therefore, to develop more efficient pest management strategies, we need more studies to lighter the geographical variation in resistance to insecticides.

## CONFLICT OF INTEREST

The authors declare no competing interest.

## AUTHOR CONTRIBUTION

**Aigi Margus:** Data curation (lead); Formal analysis (lead); Investigation (equal); Methodology (equal); Visualization (equal); Writing‐original draft (lead); Writing‐review & editing (equal). **Saija Piiroinen:** Conceptualization (lead); Formal analysis (equal); Investigation (equal); Methodology (equal); Validation (equal); Visualization (equal); Writing‐review & editing (equal). **Philipp Lehmann:** Conceptualization (equal); Formal analysis (equal); Investigation (equal); Methodology (equal); Validation (equal); Visualization (equal); Writing‐review & editing (equal). **Alessandro Grapputo:** Conceptualization (equal); Formal analysis (equal); Investigation (equal); Methodology (equal); Supervision (equal); Writing‐review & editing (equal). **Leona Gilbert:** Methodology (supporting); Validation (supporting); Writing‐review & editing (supporting). **Yolanda Chen:** Resources (equal); Writing‐review & editing (equal). **Leena Lindström:** Conceptualization (lead); Data curation (supporting); Funding acquisition (lead); Investigation (supporting); Methodology (equal); Project administration (equal); Resources (equal); Supervision (equal); Writing‐review & editing (equal).

## Supporting information

Supinfo S1

## Data Availability

Bioassay, AChE enzyme activity, gene expression, and gene sequence data for this study are available at University of Jyväskylä Digital Repository (JYX https://jyx.jyu.fi/handle/123456789/78053). https://doi.org/10.17011/jyx/dataset/78053.
