# Supplementary material for: Sequence variation and regulatory variation in acetylcholinesterase genes contribute to insecticide resistance in different populations of Leptinotarsa decemlineata
Source: Ecol Evol. 2021 Nov 1;11(22):15995–6005. doi: 10.1002/ece3.8269 (PMC8601895; doi:10.1002/ece3.8269)
Supplement: Supplementary file 1 — Supinfo S1 [file ECE3-11-15995-s001.docx]

**SUPPLEMENTARY TABLES AND FIGURES**

**Supplementary table 1.** Primers used in qPCR to analyse the expression of the Colorado potato beetle acetylcholinesterase genes (*Ldace1* and *Ldace2*).

| Gene name | Common name | Forward primer sequence (5´ - 3´)  Reverse primer sequence (5´ - 3´) | Reference |  |
| --- | --- | --- | --- | --- |
| Target genes | |  |  |  |
| *Ldace1* | Acetylcholinesterase 1 | F: CGCCGAGTTACAAAATACCC  R: TAGCGTTTCCATCCAATTCC | Revuelta *et al.* 2011 |  |
| *Ldace2* | Acetylcholinesterase 2 | F: CGTAAGGTGATGGACTGTATGAGA  R: CTTCATCGTGATTACTCCCCA | Zhu & Clark 1995 |  |
| Reference genes | |  |  |  |
| *FOXO* | Forkhead transcription factor | F: TCATGGCTATCGAGGATTCA  R: CCATTGGTGCTGTCTTGAAA | Lehmann *et al.* 2014, Kumar *et al.* 2014 |  |
| *L13e* | Ribosomal protein L13e | F: TATTCACCAGCCATCCATCA  R: GCGTCCTTCACTCTCTTTGC | Yocum *et al.* 2009 |  |

**Supplementary table 2.** Primers used for *Ldace1* and *Ldace2* gene sequencing. F- forward, R- reverse primer

| Gene | Name | F/R | 5´ - 3´ sequence |
| --- | --- | --- | --- |
| *Ldace1* | Ldace1_1F | F | ACAACATCACGCTCTTCGGT |
|  | Ldace1_1R | R | TCTAAAAGCCTGGAAGTGGTTA |
|  | Ldace2_2F | F | CGATGACAACAACGCTACGA |
|  | Ldace2_2R | R | TCAGATGGTACATGATGAAGGAA |
| *Ldace2* | LdNSf | F | GCTATACGTTGGATCAAAGACA |
|  | LdNSr | R | ACTGCTCTCATACAGTCCATCA |
|  | Ldace2_3F | F | AGCATCCATCTGATAAGCCCT |
|  | Ldace2_4R | R | CATTCATACAAGCACAGATTTATAGG |
|  | Ldace2_2F | F | GAAGCGATGAGACTCGTAA |
|  | Ldace2_1F | F | GATTGGAGATGTTGTTGGTGACTA |
|  | Ldace2_1R | R | TAGTCACCAACAACATCTCCAATC |

| a) | b) |
| --- | --- |
| c) | d) |

**Supplementary figure 1.** Percentage of survival (a,c- raw data, b, d- survival in probits) of the six populations of Colorado potato beetle after exposure to different doses (log10) of a-b) AZ and c-d) CAR insecticides.
